# Immunolocalization of Galectin-3 in Mouse Testicular Tissue

**Published:** 2011

**Authors:** Layasadat Khorsandi, Mahmoud Orazizadeh

**Affiliations:** 1 * Cell & Molecular Research Center, Faculty of Medicine, Ahvaz Jundishapur University of Medical Sciences, Ahvaz, Iran*

**Keywords:** Galectin-3, Lectin, Testis, Mice

## Abstract

**Objective(s):**

Galectin-3 (Gal-3) is a member of the ß-galactoside-binding lectins which is expressed in a variety of tissues and plays a role in diverse biological events, such as embryogenesis, adhesion, cellular proliferation, and apoptosis. In this study, the presence and distribution of galectin-3 (Gal-3) in the mouse testicular tissue was investigated.

**Materials and Methods:**

Eight adult NMRI mice were used in this study. Animals were sacrificed by decapitation under ether anesthesia. The testes were excised, fixed in 10% buffered formalin and embedded in paraffin for immunohistochemical (IHC) studies. H-score, a semi-quantitative method, was used for scaling the immunostaining.

**Results:**

Positive immunoreactivity to Gal-3 was detected in the connective tissues of the interstitium, Leydig cells and presumably peritubular myoid cells. Seminiferous tubules showed immuno-positive reaction in a stage dependent manner. Stages I-III and IX-XII of spermatogenic cycle showed mild immunostaining, while moderate immunostaining was observed at stages IV-VI. Highest immunoreactivity was observed at stages VII-VIII. H-score assessment showed a significant increase in stages of VII-VIII in comparison to other stages (*P*< 0.05).

**Conclusion:**

Expression of Gal-3 in interstitial tissue and seminiferous tubules indicated that this protein probably has multifunctional roles in the mouse testicular tissue.

## Introduction

Galectin-3 (Gal-3) is a member of the ß-galactoside-binding lectins. This protein is expressed in a variety of tissues and plays a role in diverse biological events, such as embryogenesis, angiogenesis, adhesion, cellular proliferation, apoptosis, and modulation of the inflammatory process and immune response (1). Gal-3 has also been implicated in tumor progression and metastasis in a variety of human cancers such as thyroid, pancreas, colon and breast (2-5).

Gal-3 is the most studied lectin among other members of its family. It is mainly a cytosolic molecule but can easily traverse the intracellular and the plasma membranes to reach the nucleus, mitochondria and be externalized (6-8). It has been associated with the splicing apparatus in the nucleus and so is thought to be involved in this process and perhaps the proliferative potential of the cell (9). In both the cytosol and mitochondria, it interacts with antiapoptotic signaling molecules such as Bcl-2 (10-12). 

Recently, it was reported that Gal-3 was expressed in a variety of epithelial cells of the urinary system, including the kidney (13), and in the bovine respiratory and digestive tracts during fetal development (14). Although the distribution of some glycoconjugates has been studied in boar testis (15), and also horse testis (16), little is known of the presence and distribution of galectins in the mouse testis. The aim of this study was to examine the presence of Gal-3 in the mouse testicular tissue.

## Materials and Methods

***Animals***

Eight NMRI male mice weighing 25–30 g (10-12 weeks old) were used in this study. The animals were obtained from , . The mice were housed in a temperature- and light-controlled room and fed with standard laboratory chow and water.

***Immunohistochemistry***

Animals were sacrificed by decapitation under ether anesthesia and testes from each animal were fixed in 10% formalin. After 48 hr, the testes were embedded in paraffin, sectioned (5 µm) and stained by immunohistochemistry procedure. Briefly the sections were deparaffinized and endogenous peroxidase activity was blocked by incubation in methanol containing 0.3% H_2_O_2_ for 15 min at room temperature. Antigen retrieval was performed with 10 Mm sodium citrate buffer (pH 6.0) at 98 °C for 15 min. Sections were then incubated for 1 h at room temperature with a blocking solution consisting 5% normal goat serum in PBS-A (PBS-A, 137 mM NaCl, 29 mM NaH_2_PO_4_·H_2_O, 9 mM Na_2_HPO_4_, pH 7.4). Thereafter sections were incubated overnight at 4 °C with primary antibodies, including the monoclonal antibody against Gal-3 (sc-20157, Santa Cruz Biotechnology, USA) diluted 1:100 in blocking solution. After three washes in PBS-A, sections were incubated for 30 min at room temperature with a biotinylated antimouse IgG (secondary antibody), washed in PBS-A, and then incubated with the avidin–biotin-complex (ABC Peroxidase kit, ) for a further 30 min at room temperature. Following three 5 min PBS-A washes, the antigen was finally detected by treating the sections with a 3, 30-diaminobenzidine (DAB) substrate, after which positive immunoreactivity was revealed as brown staining (17). Sections were counterstained with haematoxyline (Merck, Germany), dehydrated in 100% ethanol, cleared in xylene and mounted with Entellan permanent mounting medium (Merck, Germany). Negative controls were generated by substituting the primary antibodies with the blocking solution.

***H-score assessments***

Two observers, blinded to the immunostaining, analyzed the sections independently. From each mouse a minimum of four sections were examined. Immunostainig intensity was estimated using a semiquantitative score, H-score, method (17,18). The H-score was calculated for each section by application of the following equation: H-score = ΣPi (i+1), where i is the intensity of staining (0 ; no staining, 1 ;weak, 2 ;moderate, 3 ; strong) and Pi is the percentage of stained cells for each intensity (0 to 100%). For each mouse, at least 10 tubules/ stage were used. The stages were identified according to the criteria proposed by Russell *et al* (1990) for paraffin sections. This method provides 12 stages of the spermatogenic cycle in mice (19).

***Statistics***

The data were analyzed using Kruskal-Wallis and (non-parametric counterpart of one-way ANOVA) and followed by Mann-Whitney U test (20) for *host-hoc* analysis and were presented as mean±SD. *P*< 0.05 was considered statistically significant.

## Results

**Figure 1.**
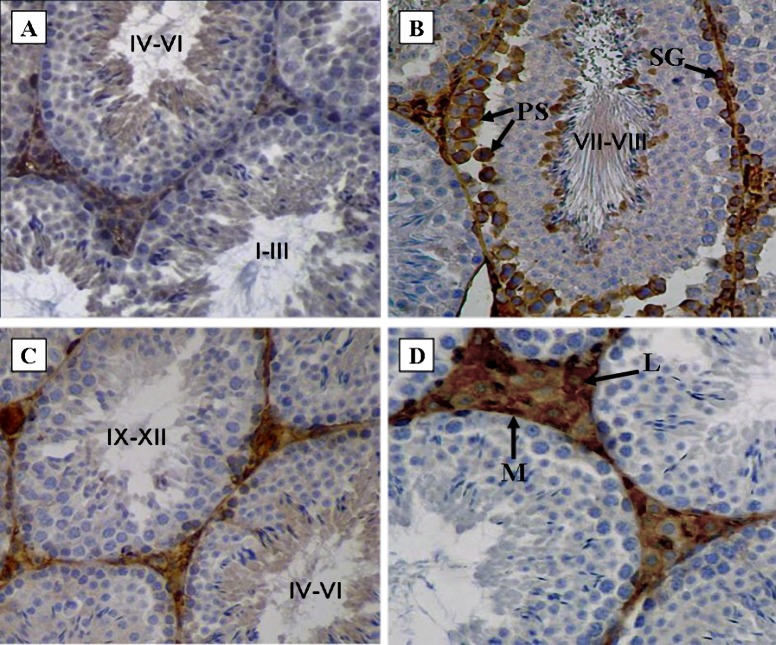
Immunohistochemical features of Gal-3 expression in the mouse testicular tissue. A, Immunostaining in stages I-III and IV-VI. B, Immunostaining in stages VII-VIII. Primary spermatocyte (PS) and spermatogonia (SG) showed strong immunoreactivity. C, Immunostaining in stages IX-XII and IV-VI. D, Immunoreactivity in interstitial tissue, Leydig cells (L) and myoid cells (M) are immuno-positive (immunostaining, original magnification: ×400).

**Figure 2.**
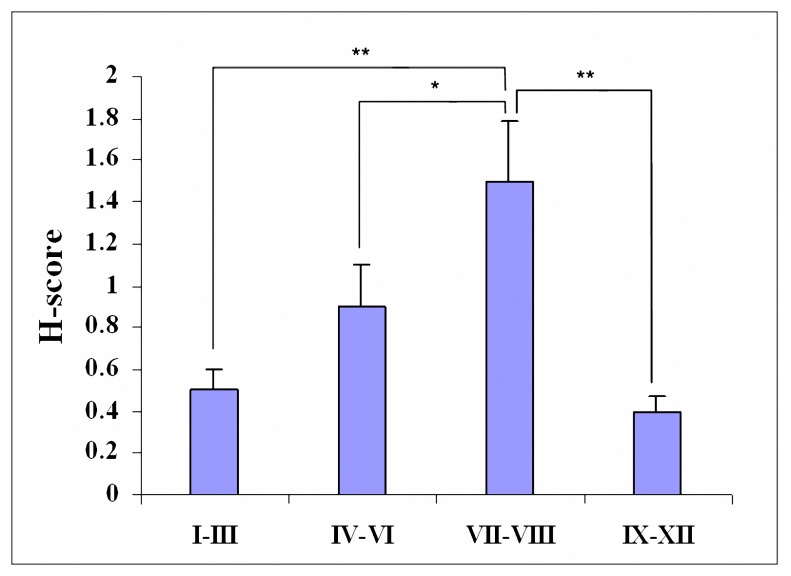
H-score assessments of Gal-3 expression in different stages of the seminiferous epithelium cycle in mice. Values are expressed as Means±SD, *P< 0.05, **P< 0.01.

In the testes, Gal-3 was immunolocalized in the interstitial tissue and seminiferous tubules. In seminiferous tubules, Gal-3 was detected in spermatogenic cells, but not in Sertoli cells. Spermatogenic cells showed various immunostaining in different stages of spermatogenic cycle. Most immonoreactivity was observed at stages VII-VIII of spermatogenic cycle. In these stages spermatogonia showed strong immunostaining. Moderate or mild immunoreactivity was seen in stages of IV-VI. Other stages showed mild immunostaining (Figures 1-A, 1-B and 1-C). The results of H-score assessments of Gal-3 expression in different stages of spermatogenic cycle are reported in Figure 2. Connective tissues in the interstitium showed immunepositive reactivity. Gal-3 was weakly detected in some cells which were identical to peritubular myoid cells (H-score: 1.79). Leydig cells showed intense immunoreactivity (H-score: 2.13). 

Immunolocalization of Gal-3 in interstitial tissue is shown in Figure 1. 

## Discussion

This is the first study to show that galectin-3 is differentially expressed in the mouse testis. Galectin-3 has previously been detected in pig, rat and human Sertoli cells (21), as well as in human and boar interstitial cells (22,23). Kim *et al* (2008) demonstrated that galectin-3 is present in the testis and epididymis of immature and mature bulls (24).

In this study we demonstrated that Gal-3 is expressed in interstitial tissue and seminiferous tubules. The pattern of Gal-3 expression was stage dependent, and stages of VII-VIII showed the highest immunoreactivity. Many studies indicated that these stages are susceptible to apoptosis (25). In previous studies we showed that dexamethasone significantly increased mouse testicular germ cells apoptosis at stages of VII-VIII (26,27). Thus, Gal-3 may relate to apoptosis in testicular tissue. Many researches have revealed that galectin-3 induces expression of Bcl-2, an important antiapoptosis factor in different tissues (10-12).

Gal-3 has diverse functions depending on whether it is expressed extracellularly or intracellularly (28). Deschildre *et al* showed that Gal-3 is exclusively located in the Sertoli cells cytoplasm of the normal human testes. They also have shown that Gal-3 has a nuclear localization in the infertile testes of human beings or rats (21). Extracellular Gal-3 is reported to induce the apoptosis of T cells (28), whereas intracellular Gal-3 has anti-apoptotic activity (12). In addition, Gal-3 on the surface of cells, such as macrophages and cancer cells is involved in laminin-mediated intercellular adhesion (29). Presence of galectin-3 in cytoplasm of spermatogenic cells, which was revealed in this study, may serve as an inhibitory mechanism to prevent germ cell apoptosis induced by environment toxic agents or alterations in testosterone production. 

Presence of Gal-3 in the interstitial tissue, which was revealed in this study, may serve as an extracellular matrix. Leydig cells also showed Gal-3 in their cytoplasm. Intratesticular androgens, secreted by Leydig cells, play an important role in spermatogenesis (30). Spermatogenesis is an elaborate process of germ cell proliferation and differentiation that leads to the production and release of spermatozoa from the testis. This complex process is dependent upon hormonal stimulation as well as dynamic interactions between the Sertoli cells and the germ cells of the seminiferous epithelium (31,32). The complexity of the intratesticular events highlights the importance of regulatory mechanisms and interactions. 

## Conclusion

The regulated expression of Gal-3 during the spermatogenic cycle suggests that this lectin is involved in reproductive biology. Further experiments are needed to clarify the mechanisms of Gal-3 in male reproduction.
